# From chloroquine to artemether-lumefantrine: the process of drug policy change in Zambia

**DOI:** 10.1186/1475-2875-7-25

**Published:** 2008-01-29

**Authors:** Naawa Sipilanyambe, Jonathon L Simon, Pascalina Chanda, Peter Olumese, Robert W Snow, Davidson H Hamer

**Affiliations:** 1 Department of Community Medicine, University of Zambia, RW 0001, Lusaka, Zambia; 2 Center for International Health and Development, Boston University School of Public Health, 5^th ^floor, 85 East Concord St., Boston, MA, USA; 3 National Malaria Control Centre, Ministry of Health PO Box 32509, Lusaka, Zambia; 4 Global Malaria Programmeme, World Health Organization, Geneva, Switzerland; 5 Malaria Public Health & Epidemiology Group, Centre for Geographic Medicine Research-Coast, Kenya Medical Research Institute/Wellcome Trust Research Programmeme, 00100 GPO, PO Box 43640, Nairobi, Kenya; 6 Centre for Tropical Medicine, University of Oxford, John Radcliffe Hospital, Headington, Oxford, OX3 9DU, UK; 7 Section of Infectious Diseases, Department of Medicine, Boston University School of Medicine, 5^th ^floor, 85 East Concord St., Boston, MA, USA

## Abstract

**Background:**

Following the recognition that morbidity and mortality due to malaria had dramatically increased in the last three decades, in 2002 the government of Zambia reviewed its efforts to prevent and treat malaria. Convincing evidence of the failing efficacy of chloroquine resulted in the initiation of a process that eventually led to the development and implementation of a new national drug policy based on artemisinin-based combination therapy (ACT).

**Methods:**

All published and unpublished documented evidence dealing with the antimalarial drug policy change was reviewed. These data were supplemented by the authors' observations of the policy change process. The information has been structured to capture the timing of events, the challenges encountered, and the resolutions reached in order to achieve implementation of the new treatment policy.

**Results:**

A decision was made to change national drug policy to artemether-lumefantrine (AL) in the first quarter of 2002, with a formal announcement made in October 2002. During this period, efforts were undertaken to identify funding for the procurement of AL and to develop new malaria treatment guidelines, training materials, and plans for implementation of the policy. In order to avoid a delay in implementation, the policy change decision required a formal adoption within existing legislation. Starting with donated drug, a phased deployment of AL began in January 2003 with initial use in seven districts followed by scaling up to 28 districts in the second half of 2003 and then to all 72 districts countrywide in early 2004.

**Conclusion:**

Drug policy changes are not without difficulties and demand a sustained international financing strategy for them to succeed. The Zambian experience demonstrates the need for a harmonized national consensus among many stakeholders and a political commitment to ensure that new policies are translated into practice quickly. To guarantee effective policies requires more effort and recognition that this becomes a health system and not a drug issue. This case study attempts to document the successful experience of change to ACT in Zambia and provides a realistic overview of some of the painful experiences and important lessons learnt.

## Introduction

Over 40 countries in sub-Saharan Africa have recently revised their national uncomplicated malaria treatment policies replacing either chloroquine (CQ) or sulphadoxine-pyrimethamine (SP) with artemisinin-based combination therapy (ACT) [1]. These policy changes have been made in response to growing evidence of the adverse consequences of malaria treatment failures [2-4] and the need to limit the future spread of drug resistance [5].

Between the late 1970s and the late 1990s, morbidity and mortality attributable to malaria rose dramatically in Zambia with incidence rates nearly tripling from 1976 to 1999 (121.5 cases/1,000 persons/year in 1976 vs. 308 cases/1,000 persons/year in 1999) [6]. From 1985 to 2001, the proportion of total inpatient cases attributable to malaria nearly quadrupled and the case fatality rate for hospitalized patients with malaria followed a similar pattern [6]. This prompted the Zambian government to review its malaria treatment policy. This resulted in a bold step to abandon CQ in favour of artemether-lumefantrine (AL) in 2002, thus making Zambia the first African country to adopt AL as a first line treatment policy nationwide. The decision-making processes and implementation approach are documented in this paper along with the challenges facing the policy to practice transition.

## Methods

All published and unpublished documentary evidence surrounding the antimalarial drug policy change were reviewed and the health system context within which they were made. These data were supplemented by the authors' observations of the policy change process (several of the authors were actively involved and attended many of the relevant meetings). Information has been structured to capture the timing of events, the difficulties and hurdles faced and the resolutions reached to the final implementation of a new treatment policy.

### Case description

#### Zambian context prior to policy change

Since the late 1970's, there was a significant increase in malaria-related morbidity and mortality seen at health facilities nationwide [6]. Direct attribution of causes for the resurgence of the malaria burden is hard to define, but it was widely held that the disease transition was a result of a combination of economic, prevention and treatment failures. The economic crisis that began in the early seventies led to a re-prioritizing of local government expenditure, resulting in budget cuts in most sectors including health. The insecticide-spraying programme, which had been a major component of malaria control during the fifties and sixties, had to re-deploy spray men to other functions or lay them off altogether. The end of the global malaria eradication campaign and the WHO's recommendation for worldwide cessation of DDT use in 1973 led to DDT being banned from use in IRS in Zambia. This dealt a final blow to the public sector malaria control programme in Zambia [6]. By the second half of the 1980s, the health budgets of the private mining companies also began to face cuts, leading to reductions in spraying coverage rates, and the mines no longer added buffer zones to their spraying programme and ceased spraying altogether in 1990. Against a background of a collapsed prevention programme and declining health sector financing the human immunodeficiency virus/acquired immunodeficiency syndrome (HIV/AIDS) epidemic grew rapidly in Zambia [7]. As with other southern African countries facing similar collapses in the health sector financing and a growing health system burden posed by the HIV epidemic, the emergence of resistance to CQ in Zambia represented a public health disaster.

#### Drug sensitivity evidence from surveillance data

Nineteen studies examined the efficacy of CQ at sentinel sites across Zambia between 1995 and 2000 using the World Health Organization (WHO) 14-day efficacy protocol [8]. In 1995 treatment failure rates (combined early and late treatment failure) in children under the age of 5 years ranged from 5.4% to 13.6% at 8 sites. By 1999, the failure rates had risen to between 24.1% and 44.0% across 10 sites [6]. Between 1995 and 1999 day 14 treatment failure rates with SP, the second line drug for uncomplicated malaria at the time, were low (0–4.2%). Two studies evaluating the efficacy of CQ were undertaken between 1999 and 2002 at six sites documented day 14 treatment failure rates were between 25.9% and 52.0% [9]. Corresponding day 14 treatment failure rates for SP during this period were between 3% and 7%. After the studies in 2002, a decision was made, based on ethical reasons, to halt further efficacy testing of CQ.

#### The policy change process

**Figure 1:**
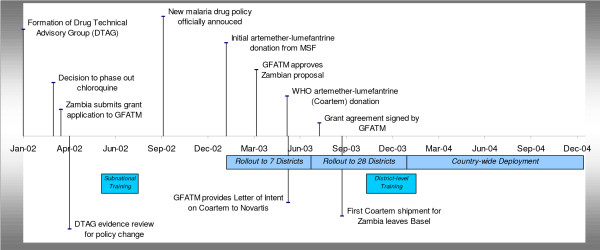
Timeline of the malaria drug policy change process in Zambia.

In recognition of the complexity of changing first-line therapy for malaria, a multidisciplinary Drug Technical Advisory Group (DTAG), a sub-group of the National Case Management Working Group with representation from the pharmaceutical, medical, research, policy and District Health Management Teams (DHMT) was established in the first quarter of 2002 (Figure 1). In addition, multiple external technical missions to the programme conducted by partners such as the WHO RBM programme and the United States Agency for International Development (USAID) provided technical support to the DTAG. The DTAG had two major tasks: a) developing the technical framework for treatment policy change; and b) designing an advocacy strategy for the new treatment policy including resource mobilization (financing for the policy change and funds for procurement of the antimalarials, among others). The DTAG provided feedback and reported to the National Malaria Taskforce and the Minister of Health. In addition to improving the malaria control programme, this policy change process was expected to strengthen the performance of other key health sector support functions such as procurement and supply, drug regulation, communications and health management information systems.

In April 2002, the DTAG reviewed the scientific evidence for policy change [10]. The evidence included the rising morbidity and mortality statistics, the CQ and SP drug efficacy studies and research evidence from neighbouring countries, notably in South Africa [11]. At this point in time, there had been no clinical efficacy studies of possible ACT replacements undertaken in Zambia as the only drugs that had been studied in Zambia up until then were CQ and SP. CQ resistance was approximately 60% (20–80% depending on site), well beyond the WHO's recommendations for changing national malaria drug policy. The DTAG concluded that immediate action was required to replace CQ as first line therapy and increase access to curative services and strengthening the health system's ability to deliver appropriate care. As such the policy addressed issues related to laboratory diagnosis, logistics and supply chain management, and behavioral change and communication.

The DTAG considered several factors regarding the replacement options to CQ, including the expected pace of resistance emergence, tolerability, safety, and procurement costs. Monotherapy options were considered. For example, SP was considered an easy option because it was already in use as the second line antimalarial and could be administered as a single dose treatment with a relatively high safety profile in all age groups. However, the DTAG were persuaded that SP was not a long-term option because parasitological resistance was already appearing in parts of Zambia [9,12] and the arguments for combined therapy were important to the DTAG in an effort to delay the future emergence of resistance. At the time of the policy change, SP resistance was low [12] but due to the fact that this drug was the treatment of choice for children weighing less than 10 kg and pregnant women, the DTAG decided to preserve the use of the drug. Additionally, SP is not truly combination therapy and hence it was not a good choice for the replacement of CQ. Amodiaquine was not considered as a serious contender as a replacement therapy, either as a monotherapy option or in combination with artesunate, because of its structural similarity to CQ and the potential for cross-resistance. Halofantrine and mefloquine were considered but due to safety concerns [13], given the likelihood of substantial unsupervised use, these drugs were not selected. Quinine was reserved for the management of severe malaria and treatment of malaria in pregnancy. By a simple process of elimination from the limited available options, in March 2002 the DTAG recommended AL for the treatment of uncomplicated falciparum malaria [14]. AL was the only fixed dose combination available at the time, had proven therapeutic efficacy even against multi-drug resistant parasites [15-17], and was also considered to have the least potential of developing drug resistance [18]. The fact that AL additionally had a comparative price advantage, offered through the differential pricing agreement between WHO and Novartis Pharma AG, made this treatment option more appealing.

Nevertheless, there remained several concerns about the adoption of ACT for Zambia. Members of the DTAG were cautious about the introduction of a new drug with limited published data on its safety profile. Four further concerns were raised by the DTAG: First, AL was at the time not recommended for use in pregnant women or children weighing less than 10 kg, two of the most vulnerable groups. Second, AL had a complex dosing schedule which included the need for a fatty meal to enhance absorption of the lumefantrine component. Third, AL had a short shelf life of 24 months. Finally, AL, despite the preferential pricing, still cost 40 times more than CQ.

It was estimated that the cost of supplying AL to cover all malaria cases in Zambia in 2002 would have exceeded the finances available for the overall Basic Healthcare Package for the entire country. Additionally, the Ministry of Health was struggling at the same time with the conflicting priority need to launch an antiretroviral therapy programme in-line with the WHO's 3 × 5 strategy [19]. Concerns were also raised about the sustainability of AL supply from a single supplier who might not scale-up production to meet the increased demand.

Despite these legitimate concerns regarding lack of previous experience with AL, financing and sustainability, alternative options were few and a definitive decision was urgently required. In October 2002, AL was officially adopted as the future first-line therapy for Zambia, a decision that received a strong political commitment from the Minister for Health.

#### Policy change preparatory phase

From October to December 2002, the DTAG, with regular support from the Minister of Health, agreed to implement the policy decision in the most expedient manner and decided upon a phased implementation plan leading to complete national coverage. The plan [14] proposed that seven early implementation districts would be the first recipients of AL and training in its use allowing lessons learnt to adapt the implementation strategy in the second phase involving 28 districts and final expansion in the third phase to the remaining 44 districts across Zambia. This ensured that once a decision had been announced action was taken, albeit in a pilot phase and allowing time for the assembly of necessary financing. At this stage, the functions of the DTAG were redefined and included two essential components: a) a case management advisory group (guidelines, training, job aids); and b) a drug transition team (procurement, forecasting, distribution, training, supervising implementation, and outcomes monitoring).

In order to fast track the policy implementation process and avoid a potentially protracted formal debate and approval process within the Zambian Parliament, and since malaria control was considered a statutory activity, it was agreed that the policy change should be reconciled within the National Health Services Act of 1995 [20]. An Information Cabinet Memorandum was prepared in order to notify the Cabinet, as per government procedure.

Further regulatory changes were required to re-register Coartem^® ^(AL) from the 4-dose regimen in the private sector to a 6-dose regimen, the WHO recommended regimen, for public sector use. A waiver was obtained through the provision in the National Pharmaceutical Act allowing for extension of a drug label. The Zambia National Formulary Committee was approached by the DTAG to endorse the use of AL and its inclusion in the Zambia National Formulary and Essential Drug List with the status of a first-line antimalarial drug. As a condition of this process, the Poisons and Pharmacy Board recommended the design of a strategy for monitoring the safety of ACTs through post-marketing surveillance, as these were relatively new drugs. The Pharmaceutical Association of Zambia in collaboration with the Poisons and Pharmacy Board were tasked to mobilize technical and financial support for this programme.

Although the introduction of health reforms in Zambia led to the decentralization of Primary Health Services in Zambia, making malaria control an integral component of the Primary Health Care package, the program also benefited from increased investments of up to $20 per capita. However, the disadvantage was that the cost of deployment of certain drugs such as AL was well beyond the budget for the package. This led to high levels of discomfort amongst the donors with fear of introducing a vertical program but nonetheless a simultaneous heightened determination for resource mobilization.

Following the necessary legislative and regulatory approvals and revisions, the immediate challenge for the DTAG was to find external financial support to complete implementation of the policy. The call for proposals in January 2002 for Round 1 of the Global Fund to Fight AIDS, Tuberculosis and Malaria (GFATM) represented a unique opportunity to secure the necessary funding to effect policy change. All elements, including a forecasted drug need, necessary to implement the drug policy change were assembled into a proposal to the GFATM. The Zambian Government submitted a four year proposal requesting 36 million US$ for malaria control from the GFATM, of which, in the first year 4 million US$ were committed for the procurement of AL and 400,000 US$ to support implementation of the new policy mostly through strengthening of laboratory malaria diagnostic capacities [14]. The application was one of a few successful first-round GFATM submissions for malaria control in Africa [21]. The GFATM decision was made in April 2003 and the funds were released to the Zambian Government in August 2003 (Figure 1).

Since the Novartis Pharma AG AL product was the only pre-qualified form of AL available at the time, a waiver had to be obtained from the Nation Tender Board to allow for single sourcing. The first order was placed and procured through the special WHO-Novartis public sector supply mechanism and the shipment arrived at the Medical Stores Limited in Lusaka, the Government's medical supply agency in September 2003 (Figure 1). The distribution, based upon monthly estimated demand by district, began in October 2003 and districts were expected to request additional stock in concert with consumption from the Medical Stores Limited. In February 2003, before the arrival of GFATM funded drugs, the non-governmental organization, Médecins Sans Frontières (MSF), donated 800,000 AL tablets to assist with the early implementation, Phase 1, seven districts. Phase 2 involved the deployment of AL to an additional 28 districts between July and December 2003. Funds to ensure the implementation of Phase 2 were made available from the flexible, government-driven, Heavily Indebted Poverty Countries (HIPC) resources and the GFATM funds. Subsequently, nationwide deployment (Phase 3) took place in the first quarter of 2004 funded largely by the funds provided by the GFATM.

The private sector played a cardinal role as 20%–30% of sick patients seek treatment from private practitioners in Zambia. In addition, local manufacturers were involved in the packaging and distribution of chloroquine. A private sector access strategy was therefore developed and funds to implement the plan were mobilized in the GFATM Round 4.

#### Policy change implementation phase

Two principal guidelines addressing malaria case management were used in Zambia prior to the malaria drug policy change: the Integrated Technical Guidelines (ITG) for front-line health workers [22] and the Integrated Management of Childhood Illness (IMCI) guidelines [23,24]. Through technical consultation between January and May 2003, a final draft of malaria-specific NMCC guideline, titled "*Guidelines for the Diagnosis and Treatment of Malaria in Zambia*", was developed to reflect the new treatment policy for uncomplicated malaria [25]. The IMCI guidelines were revised to incorporate the new drug policy and were inserted as a change into the IMCI algorithms. The ITG guideline was updated by the inclusion of an addendum in February 2003. An additional job aide was developed, with support from Novartis Pharma AG, in the form of AL treatment dosage wall charts and was distributed to the districts alongside the drug supply.

A process of cascade in-service training was initiated to introduce the final draft of the National Malaria treatment guidelines, which began with three sub-national training of trainers (TOT) trainings, each including key members of the District Health Management System Teams (DHMT). Following the sub-national training between June and July 2003, district-level training was proposed at the convenience and funding arrangements of respective DHMTs. Trainings at the district-level occurred between November 2003 and February 2004 (Figure 1). The training involved an initial orientation programme for managers, followed by a programme for referral facilities, and then two-day workshops for front-line health. The training and orientation of district health workers was synchronized with the phased AL deployment. During the training, participants were provided with individual and institutional copies of the National Malaria Treatment Guidelines [26,27,27].

A detailed information, education, and communication (IEC) strategy was developed, which included a series of TV and radio programs highlighting the policy change. Job aids, dosage charts and posters were developed and distributed to all the health facilities in the countries. The challenge was to ensure consistency in the messages particularly during the pilot phase.

## Discussion and evaluation

As part of an integrated approach for malaria control and prevention, Zambia took a bold step in 2002 and introduced ACT as part of its malaria case-management strategy in an effort to reverse the rising malaria mortality and morbidity. At this time, there was almost no experience of moving to an ACT first-line anti-malarial drug policy in Africa, with the exception of Kwa-Zulu Natal Province in South Africa [11]. The decision-making process, using data based essentially only on failure rates of currently used antimalarial drugs, was comparatively rapid, taking approximately 3 months (Figure 1). In Kenya, Uganda and Tanzania the decision to abandon CQ in favor of an equally cheap and widely used drug (SP) took several years in the late 1990's and early 2000 [28-30]. More recently in Kenya, the decision to replace failing SP with AL was quicker than the time taken to decide to replace CQ with SP, but more protracted than the decision-making process in Zambia [31]. The strong political commitment to an effective drug policy change in Zambia, notably through a direct involvement of the Minister for Health, was instrumental in arriving at an early decision in Zambia.

Moving from a policy decision in October 2002 to early implementation during the first quarter of 2003 was a remarkable achievement considering the 32 month delay to implementation in Kenya [31] and similar delays elsewhere in Africa. Several factors contributed to this rapid implementation, not least was the fortuitous timing of the GFATM funding opportunity linked to the preferential pricing arrangements for AL between the WHO and Novartis Pharma AG. The ability of the Zambian government to assemble a funding plan in time for the GFATM application was strengthened by the nature of consensus and political backing among all the Zambian stakeholders. National partners, such as MSF, were committed in 2003 to assisting Zambia's move to ACT and seed government funding through the HIPC arrangements provided some financial security to begin the process of implementation.

The preparation required to implement a new drug policy is non-trivial and here we have highlighted the legislative, regulatory and guideline revisions required in order to introduce a new antimalarial into clinical practice. These steps in policy change are often under-estimated and require considerable person-hours of discussion by under-staffed national malaria control programmes. Unlike the reluctance to implement the AL drug policy change documented in other countries from different stakeholders [31], the Zambian regulatory and pharmaceutical bodies were in broad agreement with the DTAG's decisions that created the enabling environment necessary to implement the policy change. The continued role of the DTAG from the policy decision-making through to implementation allowed for a central core throughout the whole process. The DTAG included broad representation of key stakeholders and formed a basis for a more consultative and transparent dialogue that was open to external validation.

Not all countries have adopted a phased approach to public sector introduction of AL. In Zambia, the decision to expand coverage of districts in a three-step process was not without criticism: why should district X receive AL and not district Y? However, the speed with which the implementation plan transitioned from Phase 1 to Phase 3 circumvented much of the criticism. Such a strategy also allowed the programme to build experience before national coverage, and it demonstrated that the government was committed to implementing a decision shortly thereafter to maintain confidence and credibility in the policy announcement.

Notwithstanding the comparative success of policy decision making and preparation for implementation, the rolling out of the AL drug policy change faced a number of problems. The most significant was the demand quantification, stock flow and subsequent AL ordering procedures that resulted in stock-outs at peripheral health facilities [32,33]. A particular problem for AL drug management is reconciling needs for four patient groups corresponding to four different AL pack sizes. The removal and storage of CQ also posed a challenge, although it was rarely used at the clinic level after the policy change had been announced [32]. Patients seemed more ready to accept the new treatment [34] than the health workers [32]. This is because patients experienced the rapid clearance of symptoms by the new drug while, on the part of the health workers, this drug required that they spend more time counseling the patient on how to take the doses at home.

Supply chain management for the dose specific packaging (including consumption data reporting, forecasting and storage space), expanding coverage to community levels, including the private sector in AL dispensing, assembling effective information on pharmacovigilance and ensuring better quality diagnostics and clinical management remain implementation challenges for the programme in Zambia. The AL policy change was implemented against a background of a health sector human resource crisis. Addressing the broader health system issues is critical for a successful malaria case-management strategy.

In conclusion, the transition to effective malaria case-management strategies including efficacious ACT is a fundamental cornerstone for malaria control. These policy changes are not without difficulties and demand a sustained international financing strategy for them to succeed. The Zambian experience demonstrates the need for a harmonized national consensus among many stakeholders and a political commitment to ensure that new policies are translated into practice quickly. To guarantee effective policies become effective practice requires more effort and recognition that this becomes a health system and not a drug issue.

## Competing interests

NS and RWS have both received remuneration for presentation of Zambian AL policy data at meetings hosted by Novartis Pharma AG in 2006. The drug manufacturer, Novartis, had no influence whatsoever; Novartis was not part of the drafting team nor did any representatives of the company review or provide comments on the final version of the manuscript.

## Authors' contributions

NS, PC and DHH collected, collated, and interpreted the documents used in this paper; prepared initial drafts and assisted with revisions of the manuscript; RWS, PO and JS reviewed information on elements of the policy implementation process, context and assisted in revising the manuscript.
